# Supplementary material for: Prediction of Prostate Cancer Biochemical and Clinical Recurrence Is Improved by IHC-Assisted Grading Using Appl1, Sortilin and Syndecan-1
Source: Cancers (Basel). 2023 Jun 16;15(12):3215. doi: 10.3390/cancers15123215 (PMC10296524; doi:10.3390/cancers15123215)
Supplement: Supplementary file 1 [file cancers-15-03215-s001.zip › cancers-2433883-supplementary.pdf]

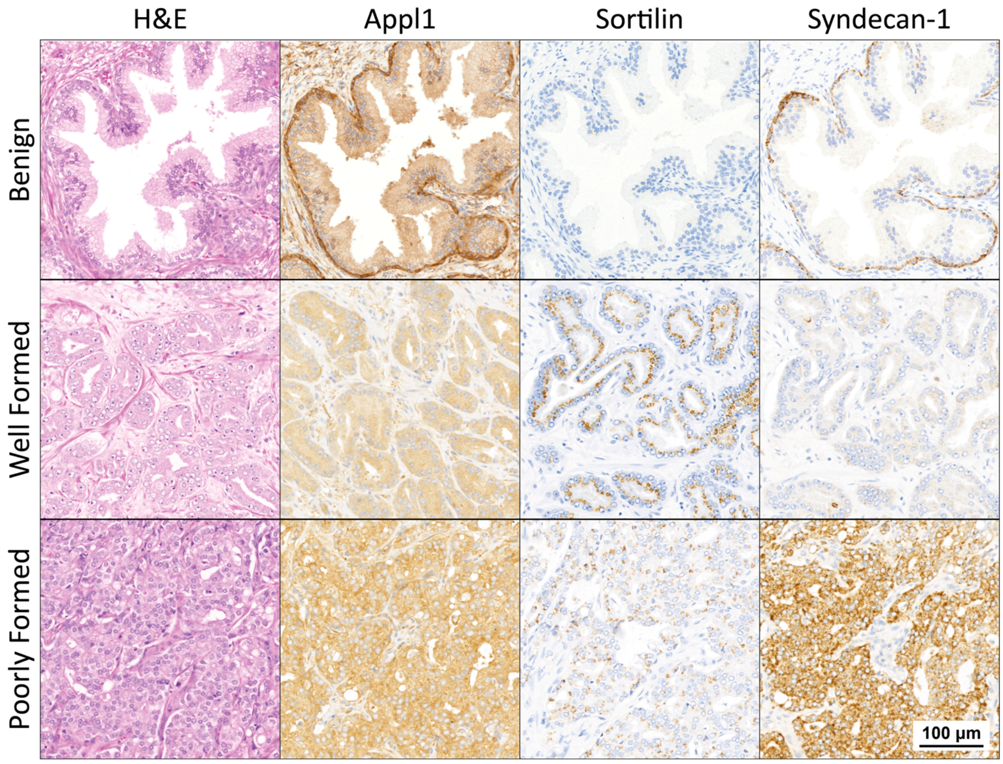

Supplementary Figure S1: Immunolabelling of Appl1, Sortilin and Syndecan-1 in well-formed and poorly-formed glands.
Serial sections of tissue from patients with prostate cancer were stained with routine H&E (first column), or labelled with Appl1 (second column), Sortilin (third column) or Syndecan-1 (fourth column). Immunolabelling with Appl1, Sortilin and Syndecan-1 is depicted by a brown immunoprecipitate (DAB). The morphologies identified include regions of benign prostatic glands, well-formed and poorly formed malignant glands.

Supplementary Table S1: Summary of patient characteristics

	**Total No. 114**
H&E, grade group	
Benign	4 (4%)
1	47 (41%)
2	27 (24%)
3	21 (18%)
4	12 (11%)
5	3 (3%)
IHC-assisted grade group	
Benign	3 (3%)
1	34 (30%)
2	36 (32%)
3	22 (19%)
4	15 (13%)
5	4 (4%)

Extracapsular extension	63 (55%)
Positive surgical margins	43 (38%)
Seminal vesicle involvement	16 (14%)
Perineural involvement	104 (91%)
Lymphovascular involvement	17 (15%)

Data are number of patients (%).

Supplementary Table S2: Summary of patient characteristics by IHC-assisted grade group

	Total<br>No. 114	Benign<br>No. 3	1<br>No. 34	2<br>No. 36	3<br>No. 22	4<br>No. 15	5<br>No. 4
H&E, grade group							
Benign	4 (4%)	2 (67%)	1 (3%)	1 (3%)	0 (0%)	0 (0%)	0 (0%)
1	47 (41%)	1 (33%)	26 (76%)	15 (42%)	4 (18%)	1 (7%)	0 (0%)
2	27 (24%)	0 (0%)	6 (18%)	17 (47%)	4 (18%)	0 (0%)	0 (0%)
3	21 (18%)	0 (0%)	1 (3%)	3 (8%)	11 (50%)	5 (33%)	1 (25%)
4	12 (11%)	0 (0%)	0 (0%)	0 (0%)	1 (5%)	9 (60%)	2 (50%)
5	3 (3%)	0 (0%)	0 (0%)	0 (0%)	2 (9%)	0 (0%)	1 (25%)

Data are number of patients (%).
